# A Synthetic Podophyllotoxin Derivative Exerts Anti-Cancer Effects by Inducing Mitotic Arrest and Pro-Apoptotic ER Stress in Lung Cancer Preclinical Models

**DOI:** 10.1371/journal.pone.0062082

**Published:** 2013-04-30

**Authors:** Jia-Yang Chen, Yen-An Tang, Wen-Shan Li, Yu-Ching Chiou, Jiunn-Min Shieh, Yi-Ching Wang

**Affiliations:** 1 Institute of Basic Medical Sciences, National Cheng Kung University, Tainan, Taiwan, R.O.C; 2 Institute of Chemistry, Academia Sinica, Taipei, Taiwan, R.O.C; 3 Department of Chemistry, National Taiwan Normal University, Taipei, Taiwan, R.O.C; 4 Division of Chest Medicine, Department of Internal Medicine, Chi Mei Medical Center, Tainan, Taiwan, R.O.C; 5 The Center of General Education, Chia Nan University of Pharmacy & Science, Tainan, Taiwan, R.O.C; 6 Department of Pharmacology, National Cheng Kung University, Tainan, Taiwan, R.O.C; Cincinnati Children's Hospital Medical Center, United States of America

## Abstract

Some potent chemotherapy drugs including tubulin-binding agents had been developed from nature plants, such as podophyllotoxin and paclitaxel. However, poor cytotoxic selectivity, serious side-effects, and limited effectiveness are still the major concerns in their therapeutic application. We developed a fully synthetic podophyllotoxin derivative named Ching001 and investigated its anti-tumor growth effects and mechanisms in lung cancer preclinical models. Ching001 showed a selective cytotoxicity to different lung cancer cell lines but not to normal lung cells. Ching001 inhibited the polymerization of microtubule resulting in mitotic arrest as evident by the accumulation of mitosis-related proteins, survivin and aurora B, thereby leading to DNA damage and apoptosis. Ching001 also activated pro-apoptotic ER stress signaling pathway. Intraperitoneal injection of 2 mg/kg Ching001 significantly inhibited the tumor growth of A549 xenograft, while injection of 0.2 mg/kg Ching001 decreased the lung colonization ability of A549 cells in experimental metastasis assay. These anti-tumor growth and lung colonization inhibition effects were stronger than those of paclitaxel treatment at the same dosage. The xenograft tumor tissue stains further confirmed that Ching001 induced mitosis arrest and tumor apoptosis. In addition, the hematology and biochemistry tests of blood samples as well as tissue examinations indicated that Ching001 treatment did not show apparent organ toxicities in tested animals. We provided preclinical evidence that novel synthetic microtubule inhibitor Ching001, which can trigger DNA damage and apoptosis by inducing mitotic arrest and ER stress, is a potential anti-cancer compound for further drug development.

## Introduction

Some potent chemotherapy drugs had been derived from nature plants. For example, podophyllotoxin, an active component purified from *Podophyllum peltatum* [1]–[3], is used as a standard treatment for venereal warts [4]. Podophyllotoxin inhibits microtubule polymerization leading to mitosis failure and cell cycle arrest [5]–[7]. Semisynthetic derivatives of podophyllotoxin, for example etoposide and teniposide, are currently used anti-cancer drugs for leukemia, lymphoma, glioblastoma, lung, and testicular cancers [8]. Many other anti-cancer drugs derived from natural herbs also possess the ability to inhibit microtubule dynamics [2], [9]. For example, taxol (or paclitaxel) and vinca alkaloid compounds are natural products purified from *Taxus brevifolia* or *Catharanthus roseus*, respectively. Previous studies revealed that paclitaxel and vinca alkaloid compounds can interrupt microtubule dynamics [9]–[13].

Although many tubulin binding agents had been developed, strong cytotoxicity towards normal cells, depression of bone marrow and increased risk of secondary acute myelogenous leukemia restrict their clinical efficacy [1], [14]–[16]. Thus, development of novel agents with better cytotoxic selectivity and limited side-effects is important for anti-cancer treatment. We developed a novel fully synthetic podophyllotoxin derivative with high purity and good yield named Ching001, and found that Ching001 showed strong cytotoxicity specifically in lung cancer cell lines but not in normal lung cells. Ching001 inhibited microtubule polymerization and arrested cell cycle at mitosis. Ching001 induced apoptosis through induction of DNA damage and activation of ER stress signaling. Ching001 showed tumor growth inhibition and prevent tumor colonization without apparent side-effects in xenograft models, suggesting that it could be further tested as a novel anti-tumor reagent.

## Results

### Ching001 Shows Selective Cytotoxicity Towards Cancer but not Normal Lung Cells

**Figure 1:**
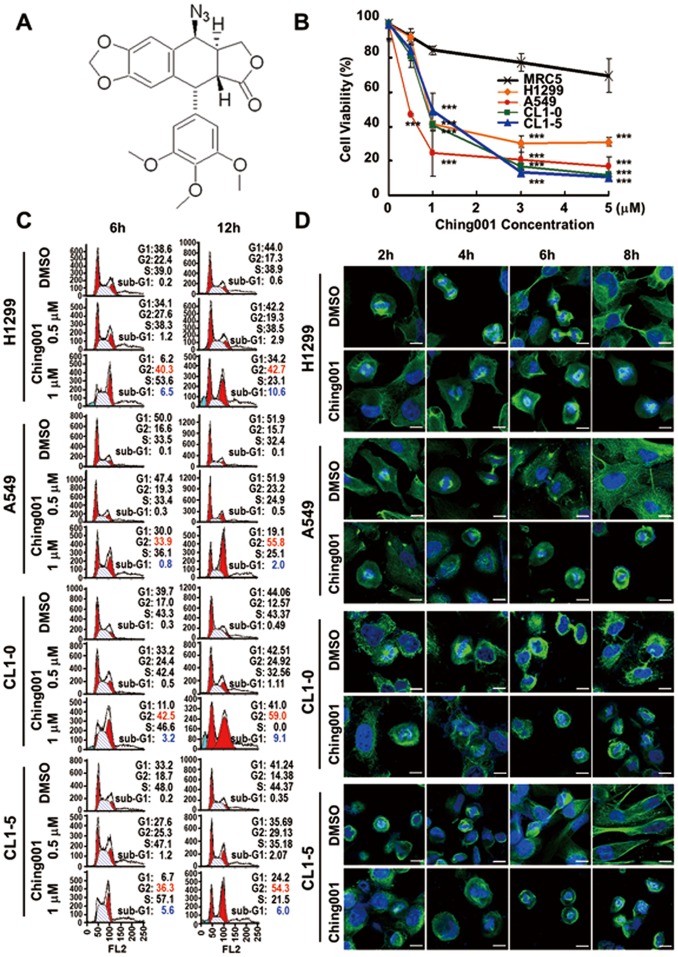
Ching001 selectively inhibits proliferation and delays M-phase progression. (**A**) The structure of Ching001 compound. (**B**) Cytotoxicity assay of normal lung MRC5 and various lung cancer cells was monitored by trypan blue staining. Cells were treated with different concentration of Ching001 for 48 h. Data represent mean ± SEM from three independent experiments. Asterisks indicate significant differences between the normal MRC5 cells and cancer cells. *** *P*<0.001. (**C**) Flow cytometry analysis of cell cycle distribution of lung cancer cell lines treated with varying doses of Ching001 and for different durations. (**D**) Immunofluorescence for α-tubulin (green) and DAPI nuclear staining (blue) after 1 µM Ching001 treatment for 2 to 8 h in S-phase synchronized lung cancer cell lines. DMSO was used as solvent control. Scale bars: 10 µm.

Ching001 is a fully synthetic compound and its structure is shown in **Fig. 1A**. The cytotoxicity of Ching001 in different lung cancer cell lines and MRC5 normal lung cell line was assayed. The IC50 calculated by regression analysis of various cell lines were: CL1-0, 1.99 µM; CL1-5, 1.90 µM; A549, 1.21 µM; H1299, 2.58 µM; and MRC5, 8.27 µM for Ching001 treatment at 48 h (**Fig. 1B**). Ching001 showed a selective cytotoxicity to lung cancer cells but not to normal human MRC5 lung cells.

### Ching001 Inhibits Microtubule Polymerization and Delays M-phase Progression

Since podophyllotoxin is known to target microtubule, its structural analogue Ching001 may also target microtubule. Microtubule assembly assay showed that 1 µM Ching001 treatment decreased the insoluble polymerized form of microtubule within 6 h (**Fig. S1A**). The immunofluorescence of α-tubulin showed significant disruption of microtubule organization in comparison with solvent control DMSO in both A549 and CL1-5 lung cancer cells (**Fig. S1B**). These results suggest that microtubule is the potential target of Ching001.

Next, we examined effect of Ching001 on cell cycle progression. Flow cytometry analyses indicated that the G2/M-phase population of the treated lung cancer cells, including H1299, A549, CL1-0, and CL1-5, increased dose-dependently with 0.5 to 1 µM Ching001 treatment for 6 h. The G2/M cell cycle population further increased dramatically, accompanied by appearance of sub-G1 fraction at prolonged Ching001 treatment for 12 h (**Fig. 1C**). Flow cytometry analysis and proliferation assay of S-phase synchronized lung cancer cells also confirmed that Ching001 treatment arrested cell cycle progression at G2/M-phase therefore inhibited proliferation (**Fig. S2**).

To further dissect the effect of Ching001 during G2/M arrest, immunofluorescence for microtubule was performed in S-phase synchronized lung cancer cells treated by Ching001 (**Fig. 1D**). The DMSO-treated cells started to enter pro-metaphase after 2 h and progressed to anaphase at 4 h after release from S phase. The cells proceeded to telophase at 6 h and reached late cytokinesis to complete mitosis at 8 h. However, Ching001-treated cells entered M-phase at 2 h and remained in metaphase even at 8 h (**Fig. 1D**).

**Figure 2:**
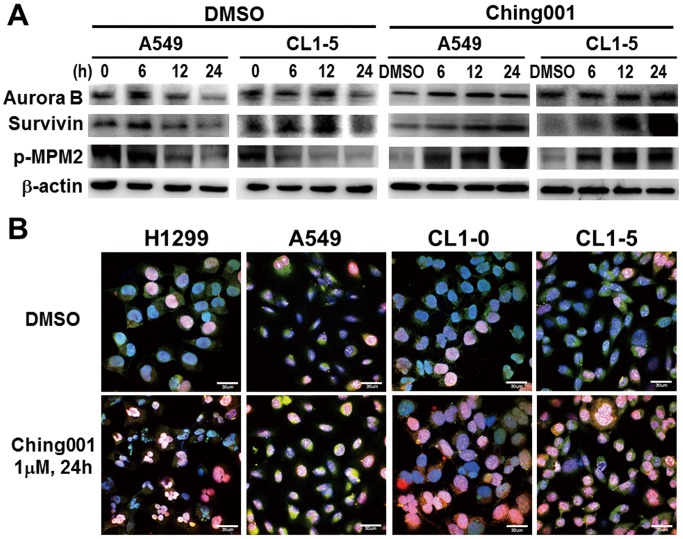
Ching001 induces M-phase arrest and DNA damage. (**A**) Western blot analyses of mitosis-related proteins after 1 µM Ching001 treatment for indicated times in lung cancer cell lines. (**B**) Dysregulation of M-phase cell cycle induced by Ching001. Lung cancer cell lines were analyzed with survivin (green), aurora B (red), and DAPI (blue) after 1 µM Ching001 treatment for 24 h. DMSO was used as solvent control. Scale bars: 30 µm.

To provide molecular evidence of M-phase arrest, we performed western blot analyses of key proteins regulating cell mitosis including aurora B, survivin and phosphorylated mitotic protein monoclonal 2 epitopes (p-MPM2) [17]–[19]. The results showed that aurora B, survivin and p-MPM2 remained high after Ching001 treatment compared with DMSO control, indicating M-phase arrest (**Fig. 2A**). In addition, immunocytochemistry staining showed that about 15% of DMSO treated cells were in M-phase by their expression of both aurora B and survivin, while Ching001 treatment significantly increased M-phase cell population which co-expressed aurora B and survivin (**Fig. 2B**). Together, these results demonstrated that Ching001 arrests cell-cycle progression at M-phase.

### Ching001-induced Mitotic Arrest Leads to DNA Damage and Apoptosis of Lung Cancer Cells

**Figure 3:**
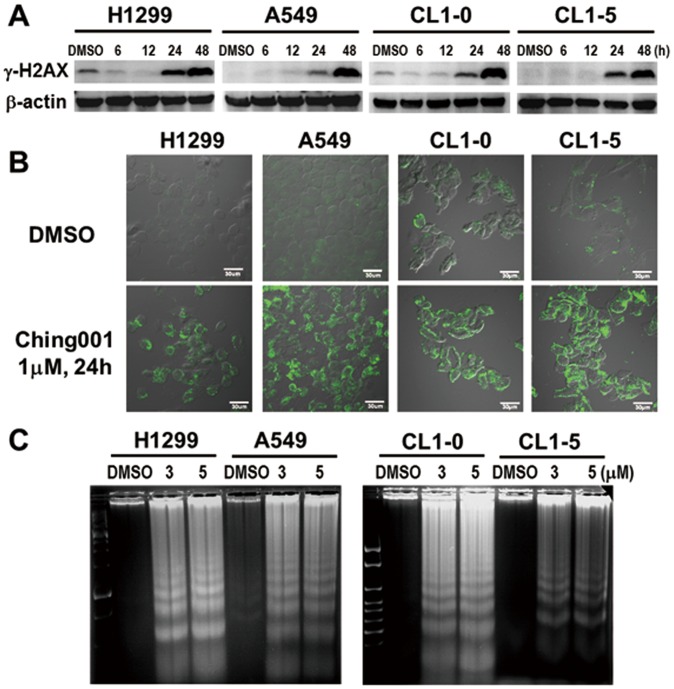
Ching001-induced mitotic arrest leads to DNA damage and apoptosis of lung cancer cells. (**A**) Western blot analyses for DNA damage marker γ–H2AX after 1 µM Ching001 treatment at indicated times. (**B**) Immunofluorescence for early-apoptotic marker PS translocation after Ching001 treatment for 24 h. Scale bars: 30 µm. (**C**) The apoptotic-specific DNA fragmentation was detected by DNA ladder analyses after Ching001 treatment for 48 h.

It had been reported that errors in mitosis generate damage and fragmentation of the DNA [20]. Our Western blot analyses showed the increase of DNA damage marker γ-H2AX in cancer cells after Ching001 treatment (**Fig. 3A**), which was confirmed by immunofluorescence for γ-H2AX (**Fig. S3**). To further examine whether the DNA damage induced by Ching001 treatment eventually results in apoptosis, PS translocation was detected by immunofluorescence after Ching001 treatment (**Fig. 3B**). In addition, apoptosis-induced DNA ladder assay was performed (**Fig. 3C**). A strong PS translocation at 24 h and induction of apoptotic DNA ladders at 48 h in Ching001-treated H1299, A549, CL1-0 and CL1-5 cells suggest that DNA damage induced by mitosis errors during Ching001 treatment eventually results in apoptosis.

### Ching001 Induces Pro-apoptotic ER Stress Signaling Pathway

**Figure 4:**
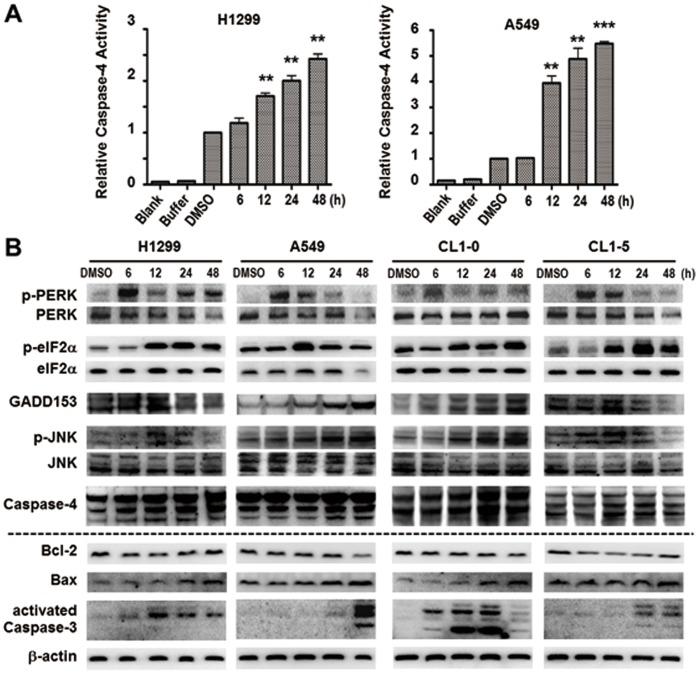
Ching001 induces ER stress mediated apoptosis. (**A**) The caspase-4 activity increased time-dependently after 1 µM Ching001 treatment in both A549 and H1299 lung cancer cell lines at indicated times. **: *P*<0.01, ***: *P*<0.001. (**B**) Western blot analyses of ER stress related signaling proteins (blots above the dot line) and apoptosis related signaling proteins (blots below the dot line) after Ching001 treatment at the indicated times.

Notably, the activity of ER stress induced caspase-4 was increased in a time-dependent manner in H1299 and A549 lung cancer cell lines after Ching001 treatment (**Fig. 4A**). To investigate the mechanisms of Ching001 treatment on pro-apoptotic ER stress induction, western blot for apoptosis regulating proteins including ER stress signaling pathways were conducted. Activation of ER stress was evident by the increased expression of p-PREK, p-eIF2α, p-JNK, GADD153 and caspase-4 after Ching001 treatment (**Fig. 4B**). In addition, Ching001 treatment decreased the expression of anti-apoptotic factor Bcl-2 and increased the expression of pro-apoptotic factor Bax, leading to apoptosis via cleavage of apoptosis executioner caspase-3 (**Fig. 4A**). These results indicated that Ching001 induced apoptosis, at least in part, via ER stress signaling pathway.

### Ching001 Exhibits *in* *vivo* Xenograft Growth Inhibition by Mitosis Arrest and Apoptosis without Induction of Apoptosis in Surrounding Tissue of Xenograft

**Figure 5:**
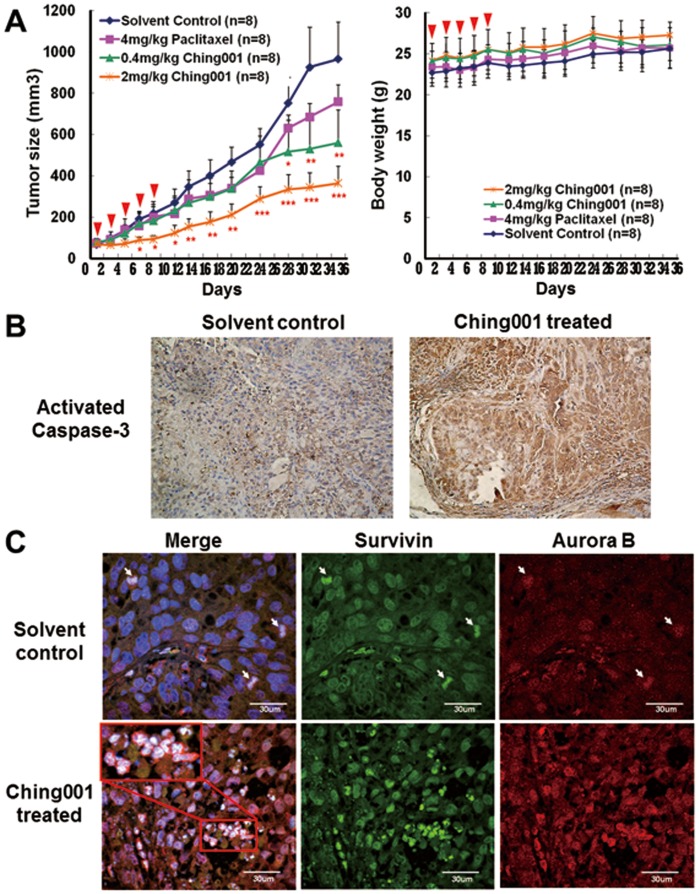
Ching001 inhibits A549 xenograft growth via M-phase arrest and apoptosis. (**A**) ICR-nude mice bearing the established A549 tumors (∼50 mm^3^) were treated with Ching001 (0.4 mg/kg or 2 mg/kg) via intraperitoneal injection on day1, day3, day5, day7, and day9 (as indicated by arrow heads). A known microtubule inhibitor, paclitaxel (4 mg/kg), was used for comparison. The tumor volumes (left) and body weight (right) were measured on every other day till day35. Points, mean; bars, ±SEM. *: *P*<0.05, **: *P*<0.01, ***: *P*<0.001. (**B**) The activated caspase-3 IHC staining of the tumor tissue of ICR-nude mice taken from solvent control group and Ching001 treatment (2 mg/kg) group. Original magnification×200. (**C**) The tissue immunofluorescence of survivin (green), aurora B (red), and DAPI (blue) of tumor xenograft from solvent control mice and Ching001 treated mice (2 mg/kg). The arrows indicated the mitotic cells in solvent control tumor. The enlarged figure represents aberrant chromosomes after Ching001 treatment. Scale bars: 30 µm.

Tumor xenograft assay was performed to test the tumor growth inhibition ability of Ching001 *in* *vivo*. Intraperitoneal injection of 0.4 mg/kg Ching001 for five times during the initial course of treatment showed the tumor growth inhibition effect, which was similar to 4 mg/kg treatment with paclitaxel, a known microtubule inhibitor (**Fig. 5A, left panel**). The tumor growth rate was further inhibited upon treatment with 2 mg/kg Ching001. No significant body weight loss in treated animals compared to control group was found (**Fig. 5A, right panel**).

To investigate whether Ching001 induced tumor apoptosis *in* *vivo*, immunohistochemistry (IHC) staining of activated caspase-3 was performed. We found an increased expression of activated caspase-3 in tumor xenografts from Ching001 treated group compared with solvent treated group (**Fig. 5B**). In addition, the Ching001 treated tumor cells appeared to have shrank in nucleus and bubbled in appearance, representing apoptotic cell death in xenograft nodule (**Fig. S4A**), while neither histological or apoptotic phenotype was observed in surrounding healthy tissue of xenograft (**Fig. S4B**). To investigate whether Ching001 induced mitosis arrest *in* *vivo*, tissue immunofluorescence of survivin and aurora B was performed. The results demonstrated an increase of cell population that co-expressed survivin and aurora B in Ching001 treated tumor tissue compared with solvent treated tissue (**Fig. 5C**). Importantly, aberrant chromosome configuration was observed in Ching001 treated xenograft (enlarged inset in **Fig. 5C**). These *in* *vivo* results corroborate with *in* *vitro* data that Ching001 induces mitosis arrest, chromosome damage, and apoptosis.

### Ching001 Inhibits Cancer Colonization Ability *in* *vivo* without Affecting Normal Vital Function

**Figure 6:**
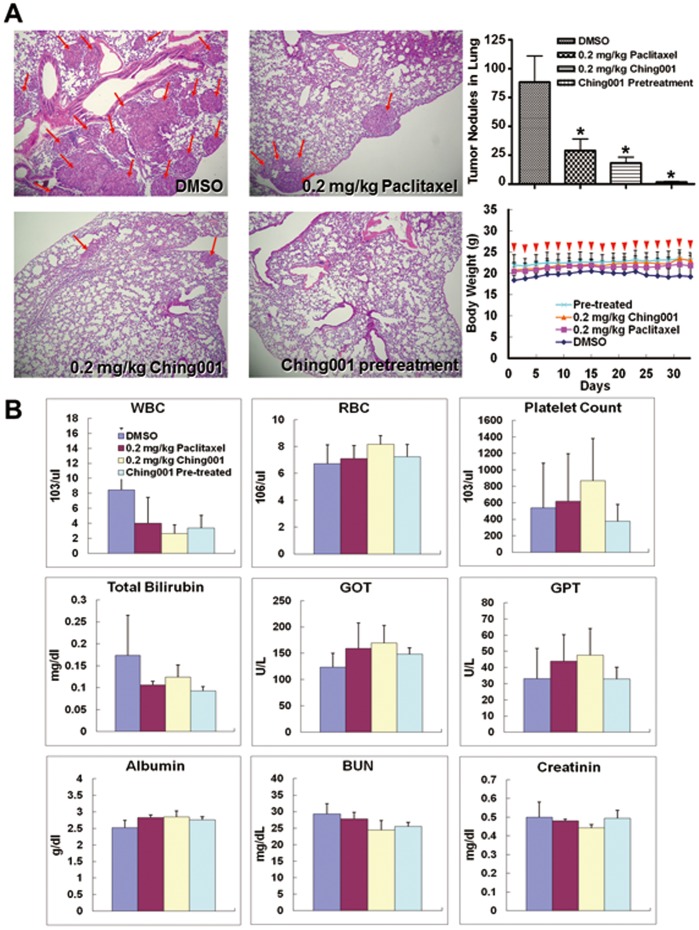
Ching001 inhibits colonization of A549 lung cancer cell in animal models without significant side effects. (**A**) A549 cells (1×10^6^) were tail vein injected into the BALB/c mice. The mice received 0.2 mg/kg Ching001 or 0.2 mg/kg Paclitaxel intraperitoneally every-other day for five weeks as indicated by arrow heads in lower right panel. Cells pretreated with 1 µM Ching001 before tail vein injection were also performed. DMSO was used as solvent control. The H&E-staining of the lung tissue (left), quantification of the tumor nodules in the lungs (upper right), and body weight (lower right) of A549 injected mice are shown. The red arrows indicated the sites of tumor nodules in the lung tissue. Original magnification×100. *: *P*<0.05. (**B**) The hematology and biochemistry tests of blood from tested mice. In the hematology tests, WBC, RBC, and platelet were tested. In biochemistry tests, GOT, GPT, albumin, and total-bilirubin are used as indicators of liver function; BUN and creatinin as indicators of renal function. The data indicated that Ching001 treatment caused no apparent change on liver and kidney functions compared to DMSO-treated animals.

To verify the *in* *vivo* colonization inhibition potential of Ching001, experimental metastasis animal studies were performed. A549 lung cancer cells were intravenously injected into the tail-vein of mice. The mice received 0.2 mg/kg Ching001, a tenth of the dosage used for anti-tumor growth animal studies, intraperitoneally every-other day from day 1 to day 35. DMSO served as solvent control and 0.2 mg/kg paclitaxel was included as a positive control. In addition, A549 cells pretreated with 1 µM Ching001 before tail-vein injection were also performed. Haematoxylin and eosin (H&E) stains showed significantly fewer tumor nodules in lungs of the mice treated intraperitoneally with Ching001 or paclitaxel compared with DMSO solvent control. Tumor nodules were seldom found in lung tissues from mice intravenously injected with Ching001 pre-treated cancer cells (**Fig. 6A, left panel**). The average number of tumor nodule in lungs was 88, 29, 18 and 2 in DMSO, 0.2 mg/kg paclitaxel treatment, 0.2 mg/kg Ching001 treatment, and Ching001 pre-treatment groups, respectively (**Fig. 6A** **, upper-right panel**). There was no significant loss in body weight in all treated animals (**Fig. 6A** **, lower-right panel**). All the biochemistry analysis of blood samples from tested animals showed no apparent adverse effects on liver and kidney functions compared with solvent control (**Fig. 6B**). In addition, the H&E staining did not show significant organ disorder in heart, kidney, liver, and lung dissected from Ching001-treated mice (**Fig. S5**). The data suggest that Ching001 treatment *in* *vitro* or *in* *vivo* inhibit lung colonization. Notably, continuous treatment of Ching001 for 35 day did not show detectable toxicity of treated animals.

## Discussion

**Figure 7:**
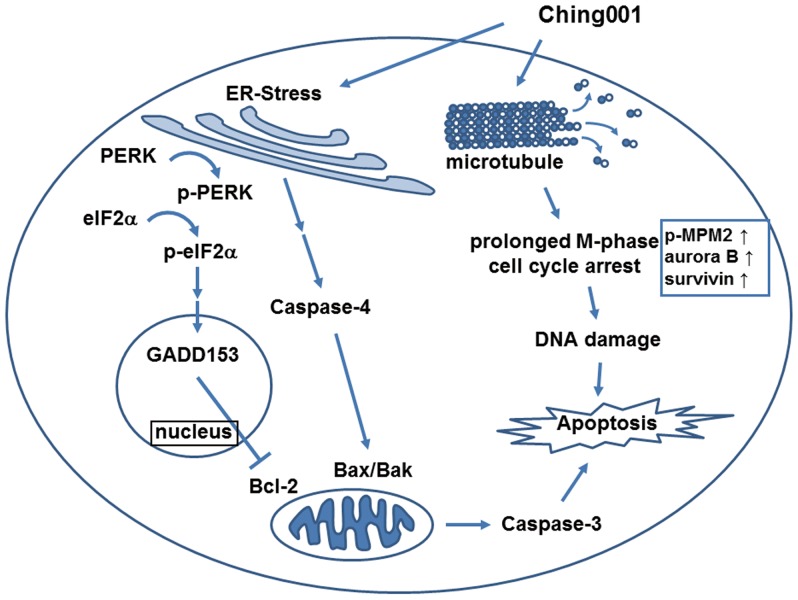
Summary of the possible anti-tumor mechanisms of Ching001. Rapid cell cycle progression is one of the features of the proliferated cancer cell. Inhibition of microtubule polymerization by Ching001 arrests M-phase cell cycle progression and leads to DNA damage. In addition, Ching001 treatment triggered activation of ER stress signaling pathway through the activation of PERK and caspase-4 signaling cascade. Prolonged M-phase arrest and activated ER stress signaling subsequently induce apoptosis in the cancer cell treated with Ching001.

To develop anti-cancer drugs with better efficacy and limited side-effects, we designed a fully synthetic compound Ching001 and examined its anti-tumor activities *in* *vitro* and *in* *vivo* in lung cancer model. Ching001 showed specific cytotoxicity against various human lung cancer cell lines at dosages in sub-micromolar range with no apparent cytotoxicity against normal human lung cell line. Animal studies showed that Ching001 inhibited tumor growth and colonization *in* *vivo* without significant side-effects in tested mice. The molecular role of Ching001 on tumor growth inhibition is mediated, at least in part, by microtubule de-polymerization leading to mitosis arrest and DNA damage. These events together with ER stress induction, eventually led to apoptosis of the lung cancer cells *in* *vitro* and *in* *vivo* (**Fig. 7**).

Our western blot analyses detected a sustained expression of p-MPM2 representing entrance of M-phase cell cycle [18], [19], indicating that Ching001 treated cells exited from G2 and preceded to M phase. In addition, aurora B and survivin control the arrangement and separation of chromosome during mitosis. Degradation of aurora B and survivin at M-phase facilitates the end of the mitosis [17]. We observed an increased co-expression of aurora B and survivin in both cell and animal samples, suggesting that Ching001 inhibits exit from M-phase. Our α-tubulin immunofluorescence of S-phase synchronized cells demonstrated a cell cycle arrest at metaphase. All together, our results support the hypothesis that Ching001 arrests cell cycle at M-phase which may be due to the inhibition of microtubule polymerization and disruption of spindle microtubule organization. Prolong M-phase arrest in cells results in failure of chromosome segregation and subsequent death from mitosis [21]. Our current cellular and animal results provide evidence that Ching001 treatment induces M-phase arrest followed by DNA damage and apoptotic cell death.

Failure of nuclear migration and division could disrupt the homeostasis of the ER [22]. ER transmembrane sensors known as PERK and IRE1α will then be activated by phosphorylation which subsequently activates the caspase-4 [23]. Ching001 treatment increased the expression of ER stress markers and apoptosis markers at 6–12 h. These results suggested that Ching001-induced cancer cell apoptosis may be caused concomitantly via induction of mitosis defects and ER stress activation at early time points. In addition, no apparent activation of intrinsic apoptosis signaling protein caspase-9 and extrinsic apoptosis signaling protein caspase-8 were observed after Ching001 treatment (data not shown), suggesting that Ching001 induced cancer cell apoptosis is via ER stress. Attenuation of ER-mediated apoptosis induced by Ching001 in ER sensors knocked down cells is worthy of further investigation.

Podophyllotoxin is the natural product analogue of Ching001 [5]–[7]. Podophyllotoxin derivative compounds used in clinical cancer treatment often show resistance due to aberrant expression and mutation of specific β-tubulin isotypes during the course of treatment [24]. In addition, paclitaxel resistance has also been reported to correlate with increased expression of p-glycoproteins [25], [26]. It is worth mentioning that Ching001 treatment showed a similar cytotoxic effect in various lung cancer cells expressing different levels of p-glycoproteins (**Fig. 1B** and **Fig. S6**). Additionally, Ching001 treatment showed a strong cytotoxic activity toward an etoposide-resistant human epidermal cancer cell line (**Fig. S7**). Whether Ching001 is a potent compound for the treatment of taxanes- or etoposide-resistant cells warrants further studies.

In conclusion, Ching001 exhibits anti-tumor growth and colonization inhibition efficacy without adverse effects in our preclinical tests. These results highlight the therapeutic potential of Ching001 in cancer treatment. Synthetic Ching001 compound of high purity and yield with cancer cell-specific cytotoxicity is a potential candidate to be tested as a lead pharmaceutical compound for cancer treatment.

## Materials and Methods

### Ethics Statement

All animals were obtained from the National Laboratory Animal Center (Republic of China, Taiwan) with the approval of Institutional Animal Care and Use Committee (IACUC), National Cheng Kung University (IACUC Approval No. 98099) and were maintained in pathogen free conditions. The study approval by the review board institution and ethics committee was confirmed by National Cheng Kung University.

### Cell Line, Cell Culture, and Synchronization

Normal human lung epithelial cell MRC5 and human lung adenocarcinoma cell lines CL1-0, and CL1-5 were obtained from Dr. P.C. Yang (Department of Internal Medicine, National Taiwan University Hospital, Taiwan) [27]. The human non-small cell lung cancer cell lines H1299 and A549 were obtained from the American Type Cell Culture. The human epidermal cancer cell lines KB and KB-7D cells were provided by Dr. Jang-Yang Chang from National Health Research Institute, Tainan, Taiwan. KB cell line was original purchased from the American Type Cell Culture and KB-7D is an etoposide-resistant cell line derived from KB cell line [28]. All cells were grown in DMEM (Gibco, Invitrogen Corporation, Carlsbad, CA) with 10% FBS (Gibco) and 1% penicillin/streptomycin (Gibco) and incubated at 37°C in 5% CO_2_ humidified atmosphere. To synchronize the cells at S-phase, cells were treated with 2 mg/ml aphidicolin (Sigma-Aldrich, St. Louis, MO) for 24 h and released into the cell cycle before Ching001 treatment.

### Compounds Used

The podophyllotoxin derivative, Ching001, was prepared by the co-author W.-S. Li. Request for the compound shall be sent to wenshan@chem.sinica.edu.tw.

### Cytotoxicity Assay

Cells (3×10^4^) were seeded on 6-well culture dishes and different concentration of Ching001 (0.5, 1, 3, 5 µM) were added to each well for 48 h. Cell number and viability were determined by trypan blue staining and cell counting.

### Microtubule Assembly Assay

Microtubule assembly assay was performed according to the previous study [29]. Cells (1×10^6^) were seeded onto 100 mm culture dishes and treated with 1 µM Ching001 or DMSO control for 6 to 48 h. The supernatant fraction of total cell lysates were collected as soluble αβ-tubulin dimers and quantified by Bradford assay. The pellet was collected as insoluble protein containing polymerized microtubules which was boiled with protein sample buffer to dissolve proteins. About 50 µg of soluble protein lysates were used as loading control, and equal volume of insoluble protein lysates were used for α-tubulin immuno-blotting. All antibodies and their reaction conditions used are listed in **Table S1**.

### Flow Cytometry

CL1-0, CL1-5, A549, and H1299 cells (1×10^6^) were collected after 6 to 12 h treatment with 0.5 µM or 1 µM Ching001, washed twice with Hank’s balanced salt solution (Sigma-Aldrich) and -20^o^C ethanol fixation overnight. Cells were incubated with DNA intercalator Propidium Iodide (Sigma-Aldrich) at 37^o^C for 1 h with 1 µg/ml RNase A and 0.1% Triton-X100. About 3×10^4^ cells were detected using FACScalibur instrument (BD Biosciences, San Jose, CA) to analyze the cell cycle distribution.

### Immunocytochemistry Staining

Cells (1×10^4^) were seeded in chamber slides and treated with Ching001 or DMSO control for 48 h. The treated cells were hybridized with α-tubulin antibody and DAPI after fixation. Antibodies of survivin and aurora B were used for mitotic cell staining. Anti-PS antibody was used for detection of early stage of apoptosis. Cells were then photographed under an OLYMPUS FV1000 confocal microscope. Detailed procedures and antibodies are described in **Table S1**.

### Western Blot Analysis

Samples containing equal amounts of protein (50 µg) were separated on a 10% SDS-PAGE and electroblotted onto Immobilon-P membranes (Millipore). Western blot was performed to measure protein expression level. Detailed procedures and antibodies are described in **Table S1**.

### DNA Ladder Assay

Cells (1×10^6^) were treated with DMSO control or 3 to 5 µM Ching001 for 24 to 48 h, DNA was extracted using DNA extraction buffer (0.2 µM phosphate-citrate buffer, pH 7.8; 37^o^C, 1 h reaction), followed by RNase A and proteinase K treatment. Agarose gel electrophoresis was used for DNA ladder analyses.

### Caspase-4 Activity Assay

Caspase-4 activity assay was performed by caspase-4 activity assay kit (GeneTex, Irvine, CA). In brief, about 200 µg of total cell lysates with DMSO control or Ching001 treatment for 6 to 48 h were used for the assay. The activity of the caspase-4 was determined by cleavage of LEVD-AFC substrate and detected by ELISA reader (Ex/Em: 400/505).

### Quantitative Reverse Transcriptase-polymerase Chain Reaction (qRT-PCR)

The mRNA expression of p-glycoprotein family genes *ABCB1* and *ABCG2* were detected by qRT-PCR in both normal and cancer lung cell lines. Total RNA was extracted from different human lung cell lines using TRIzol reagent (Invitrogen, Carlsbad, CA). About 4 µg of RNA were reverse-transcribed into cDNA using SuperScript reverse transcriptase (Invitrogen). Quantitative RT-PCR were performed to detect the mRNA expression level of target genes using the StepOnePlus™ Real-Time PCR System (Applied Biosystems, Foster City, CA) with *GAPDH* as internal control. The primers for *ABCB1* are: sense 5′-AAATTGGCTTGACAAGTTGTATATGG-3′, antisense 5′-CACCAGCATCATGAGAGGAAGTC-3′; for *ABCG2*: sense 5′-TCATCAGCCTCGATATTCCATCT-3′, antisense 5′-GGCCCGTGGAACATAAGTCTT-3′; for *GAPDH*: sense 5′- GAGTCAACGGATTTGGTCGT-3′, antisense 5′- TTGATTTTGGAGGGATCTCG-3′. The cDNA samples were amplified using the SYBR Green (Applied Biosystems) and the thermal cycling condition comprised of 95°C for 10 min followed by 45 cycles at 95°C for 3 sec and 60°C for 30 sec. Cycle threshold (Ct), the fractional cycle number at which the amount of amplified target reached a fixed threshold were determined. The normalized ratios of target genes were calculated using ΔCt [ΔCt = Ct_-target gene_ − Ct_-GAPDH_]. Data were presented as fold differences relative to IMR90 normal lung cell genes expression based on calculations of 2^−ΔΔCt^ (ΔΔCt = ΔCt_-lung cell line_ − ΔCt_-IMR90_).

### MTT Cytotoxicity Analysis

Different concentrations of Ching001 were added into each plate and incubated for 48 h. Cell viabilities under different concentrations of compound treatment were determined by 3-(4,5-dimethylthiazol-2-yl)-2,5-diphenyl tetrazolium bromide (MTT, Sigma, St. Louis, MO) assay. In the untreated control, 0.1% DMSO-containing medium was used.

### *In vivo* Tumor Xenograft Formation Assay

ICR-Foxn1 nude mice (5-week old) were acquired from the National Laboratory Animal Center with the approval of Institutional Animal Care and Use Committee (IACUC), National Cheng Kung University (IACUC Approval No. 98099), and raised in a specific pathogen free environment. A549 cells (5×10^6^) were subcutaneously injected as xenograft into mice and allowed to grow up to 50 mm^3^ tumor nodule within 2-weeks. Mice were then intraperitoneally injected with 0.4 mg/kg, or 2 mg/kg Ching001, or 4 mg/kg paclitaxel as positive control, or solvent control (ethanol:cremophore:ddH_2_O = 2∶1∶7) at day 1, 3, 5, 7, 9. The volume of the xenograft and the weight of mice were measured and quantified during 35 days of drug treatment. The xenograft volume was calculated as (length×width square)/2 in mm^3^. Major organs including heart, lung, liver, kidney, and xenograft nodule were dissected and stained with H&E for further confirmation.

### *In vivo* Experimental Metastasis Assay

The BALB/c mice were acquired and raised after obtaining appropriate institutional review board permission as described above. A549 (1×10^6^) cells were intravenously injected into tail-vein of BALB/c mice, which were then intraperitoneally given DMSO control, 0.2 mg/kg Ching001 or 0.2 mg/kg Paclitaxel every-other day for five weeks. In addition, cells pretreated with 1 µM Ching001 before tail vein injection were also performed. The colonized tumor nodules in lung tissue were dissected and stained for further confirmation.

### Immunohistochemistry (IHC) Staining

Using standard de-paraffinization and rehydration techniques, the IHC slides were stained with activated caspase-3, survivin, or aurora B antibody to detect the protein expression in lung tumor xenograft of tested mice. After hybridization with biotinylated secondary antibody and streptavidin-horseradish peroxidase reaction, the color was produced by reacting with DAB substrate (DAKO, Glostrup, Denmark). Hematoxylin was used for nuclear staining. For fluorescence staining, Alexa Fluor-488 and Alexa Fluor-546 were used as secondary antibodies, and DAPI was used for nuclear staining. The surrounding non-neoplastic stroma served as an internal control for each slide. Detailed procedures and antibodies are described in **Table S1**.

### Biochemistry and Hematology Tests

Whole blood samples of experimental mice were collected by intracardiac puncture with or without EDTA anticoagulant. Biochemistry evaluation included glutamate oxaloacetate transaminase (GOT), glutamate pyruvate transaminase (GPT), albumin levels, blood urea nitrogen (BUN), creatinine, and total-bilirubin (T-bilirubin) levels. Hematology tests included platelet count, red blood cell (RBC), and white blood cell (WBC). All experiments and procedures were done in accordance with the Institutional Care Use Committee guidelines.

### Statistical Analysis

The SPSS program (SPSS Inc., Chicago, IL) was used for all statistical analysis. Statistical analysis was performed using Student’s *t*-test. Data shown are representatives of at least three independent experiments. Data represent mean ± SEM. P<0.05 was considered to be statistically significant.

## Supporting Information

Figure S1 **Ching001 treatment inhibits microtubule polymerization.** **(A)** The microtubule assembly assay of lung cancer cell lines with 1 µM Ching001 treatment at indicated times. The insoluble proteins represent the polymerized form of microtubule, which was decreased after Ching001 treatment. The soluble proteins represent the αβ-tubulin dimer, which was not affected by Ching001 treatment. **(B)** A549 and CL1-5 were analyzed with α–tubulin (shown in green) and DAPI nuclear staining (shown in blue) after 1 µM Ching001 treatment for 24 h. DMSO was used as solvent control. Scale bars: 20 µm.
(TIF)

Figure S2 **Ching001 treatment delays G2/M phase progression and inhibits cell proliferation in synchronized lung cancer cell lines.** **(A)** Flow cytometry analysis and **(B)** proliferation assay of S-phase synchronized lung cancer cell lines by 1 µM Ching001 treatment and followed for times as indicated. DMSO was used as solvent control. M-phase arrest is indicated by arrow in the panel of 8 h post-treatment (A). The relative cell number was quantified and normalized to control group shown as a percentage in the graph (B). P values determined using two tailed t-test. Data represent mean ± s.e.m. (n = 3). *: *P*<0.05, **: *P*<0.01, ***: *P*<0.001.
(TIF)

Figure S3 **Ching001 treatment induces the expression of DNA damage marker γ-H2AX.** Immunocytochemistry staining for DNA damage marker γ–H2AX (shown in green) and DAPI for nuclear staining (shown in blue) after 3 µM Ching001 treatment for 24 h. DMSO was used as solvent control. Scale bars: 10 µm.
(TIF)

Figure S4 **H&E-stain and activated caspase-3 IHC-stain of tumor xenograft tissues and surrounding healthy tissues.** **(A)** Hematoxylin and eosin (H&E)-staining of solvent control treated xenograft tissue showed that the tumor cells grew well as a nodule (blue arrow). However, Ching001 treated xenograft tissue showed that the tumor cells in nodule had shrank in the nucleus (red circle) and bubbled in appearance between apoptotic tumor cells (red arrow). **(B)** H&E-staining (upper) and activated caspase-3 IHC-staining (lower) of surrounding healthy tissue (Normal) of solvent control treated xenograft and Ching001 treated xenograft showed that neither histological or apoptotic phenotype was observed.
(TIF)

Figure S5 **H&E-stain of major organ tissues after Ching001 treatment.** H&E staining of the major organ tissues from tested ICR-nude mice with solvent control group and Ching001 treatment group. The tissues examined included heart, kidney, liver, and lung.
(TIF)

Figure S6 **Quantitative RT-PCR analysis for relative expression of** ***ABCB1*** **and** ***ABCG2*** **genes encoding p-glycoproteins in various normal and cancer lung cell lines.** Quantitative RT-PCR showed that the mRNA expression of *ABCB1* and *ABCG2* genes encoding p-glycoproteins in H1299 and CL1-5 lung cancer cells were higher than in normal lung cells IMR90 and Beas2B.
(TIF)

Figure S7 **Cytotoxicity assay of Ching001 on etoposide-resistant human epidermal carcinoma cell lines.** The cytotoxicity of parental human epidermal carcinoma cell line KB and etoposide-resistant KB-7D cell line was evaluated with etoposide or Ching001 treatment for 48 h. KB-7D etoposide-resistant cells did not show cytotoxicity to etoposide at all doses tested. However, Ching001 exhibited strong cytotoxicity to both KB and KB-7D cells with IC50 of 0.63 µM for KB and 0.76 µM for KB-7D.
(TIF)

Table S1 **Antibodies and their reaction conditions used in the present study.**
(DOC)
