# Supplementary figures and images for: A Synthetic Podophyllotoxin Derivative Exerts Anti-Cancer Effects by Inducing Mitotic Arrest and Pro-Apoptotic ER Stress in Lung Cancer Preclinical Models
Source: PLoS One. 2013 Apr 30;8(4):e62082. doi: 10.1371/journal.pone.0062082 (PMC3639983; doi:10.1371/journal.pone.0062082)

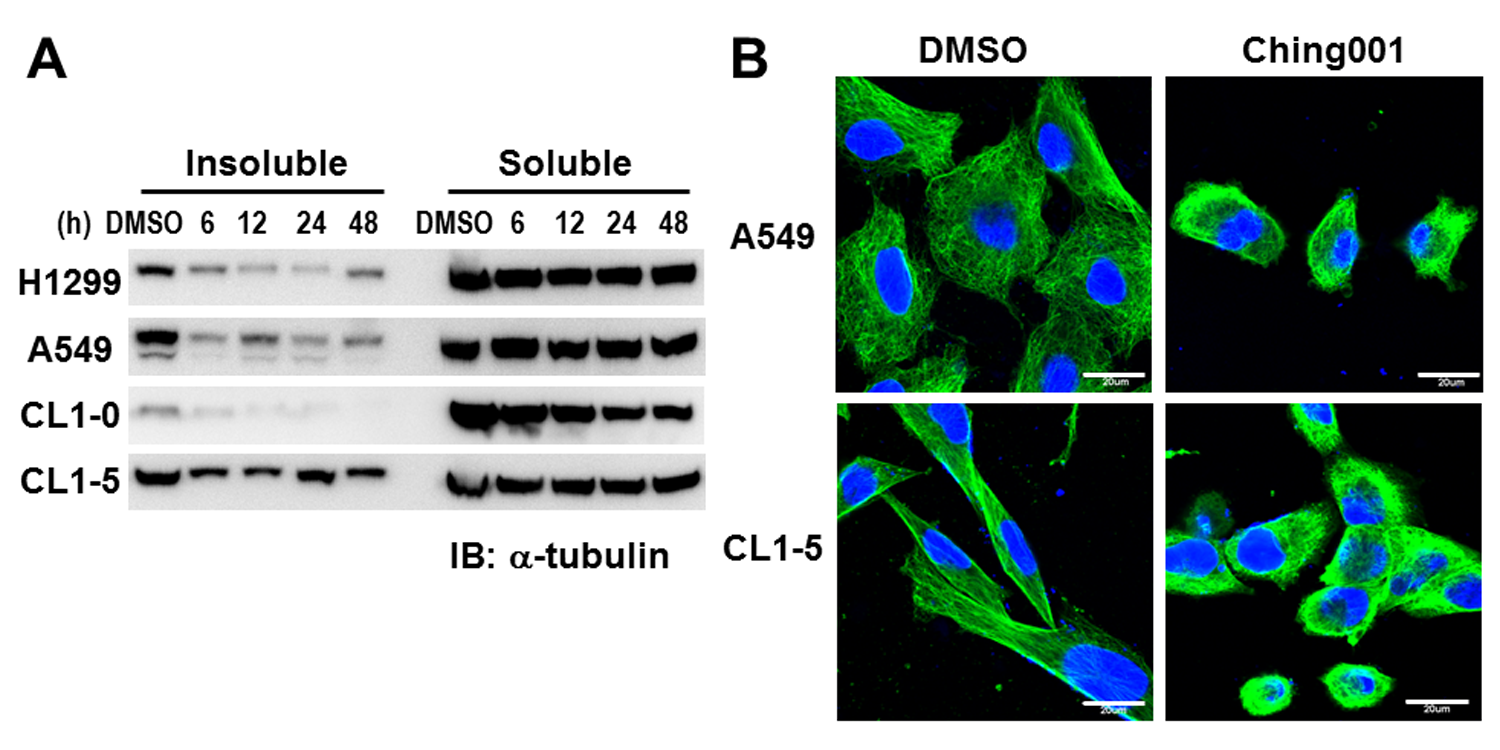

Supplement: Figure S1 — Ching001 treatment inhibits microtubule polymerization. (A) The microtubule assembly assay of lung cancer cell lines with 1 µM Ching001 treatment at indicated times. The insoluble proteins represent the polymerized form of microtubule, which was decreased after Ching001 treatment. The soluble proteins represent the αβ-tubulin dimer, which was not affected by Ching001 treatment. (B) A549 and CL1-5 were analyzed with α–tubulin (shown in green) and DAPI nuclear staining (shown in blue) after 1 µM Ching001 treatment for 24 h. DMSO was used as solvent control. Scale bars: 20 µm. (TIF) [file pone.0062082.s001.tif]

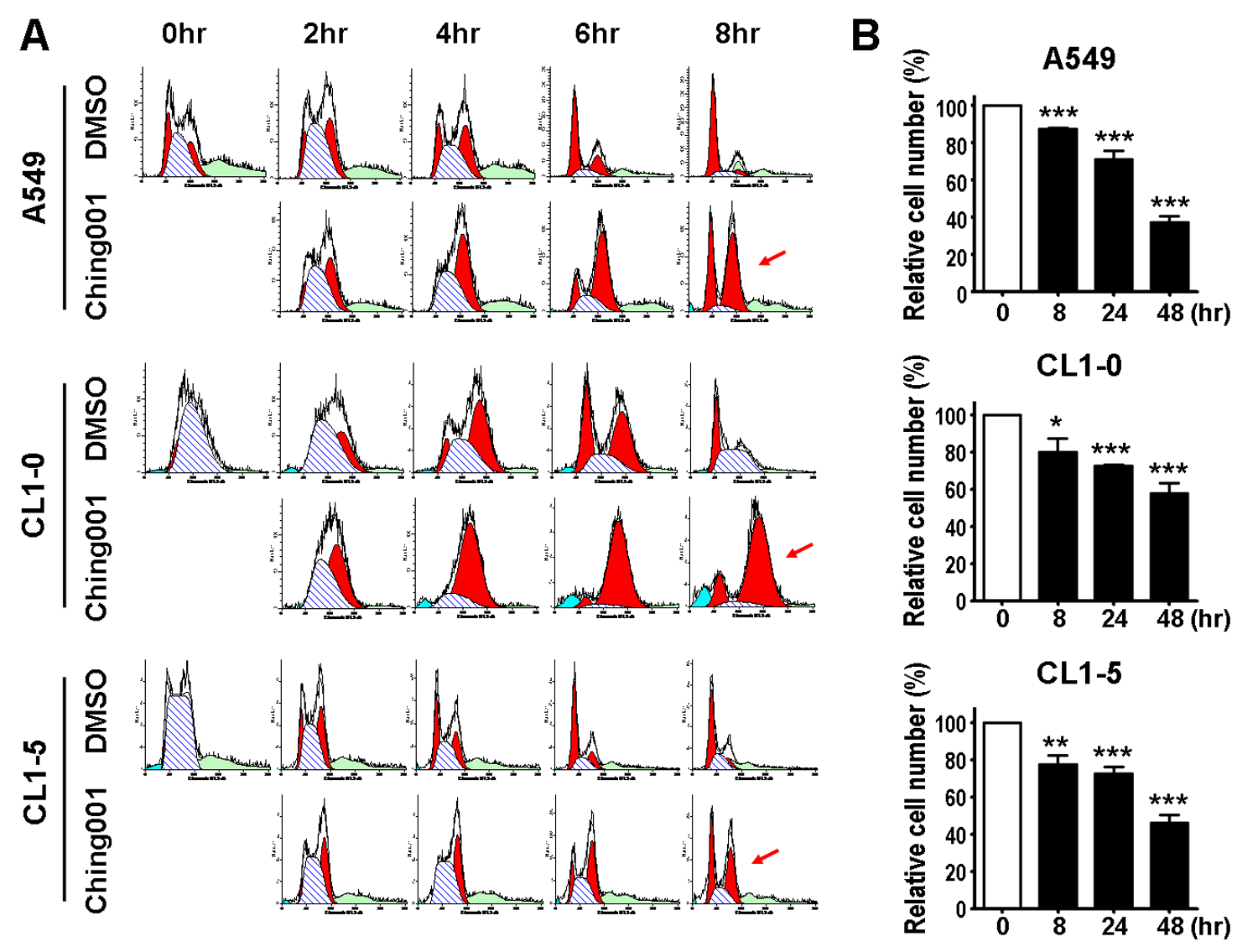

Supplement: Figure S2 — Ching001 treatment delays G2/M phase progression and inhibits cell proliferation in synchronized lung cancer cell lines. (A) Flow cytometry analysis and (B) proliferation assay of S-phase synchronized lung cancer cell lines by 1 µM Ching001 treatment and followed for times as indicated. DMSO was used as solvent control. M-phase arrest is indicated by arrow in the panel of 8 h post-treatment (A). The relative cell number was quantified and normalized to control group shown as a percentage in the graph (B). P values determined using two tailed t-test. Data represent mean ± s.e.m. (n = 3). *: P<0.05, **: P<0.01, ***: P<0.001. (TIF) [file pone.0062082.s002.tif]

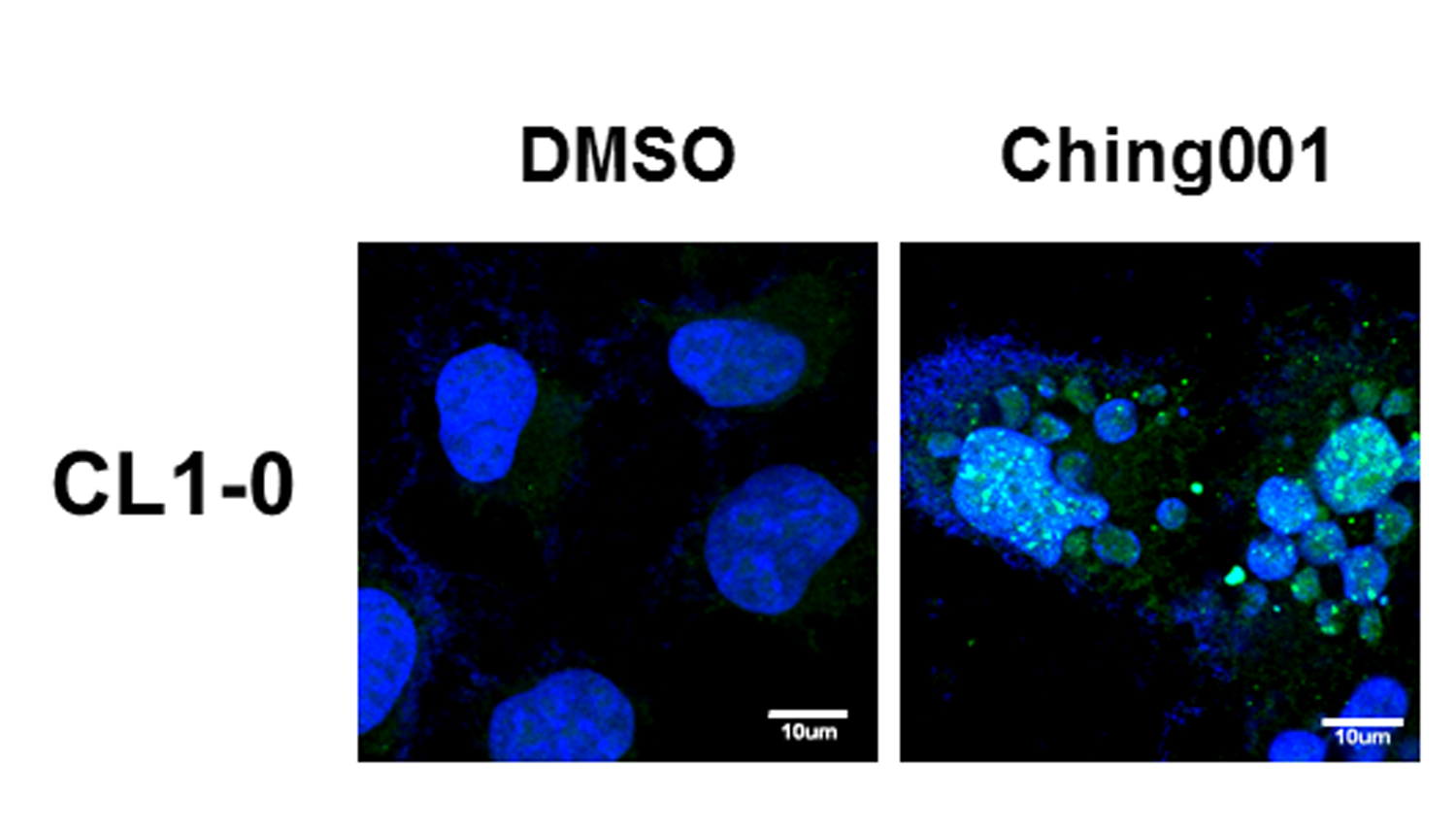

Supplement: Figure S3 — Ching001 treatment induces the expression of DNA damage marker γ-H2AX. Immunocytochemistry staining for DNA damage marker γ–H2AX (shown in green) and DAPI for nuclear staining (shown in blue) after 3 µM Ching001 treatment for 24 h. DMSO was used as solvent control. Scale bars: 10 µm. (TIF) [file pone.0062082.s003.tif]

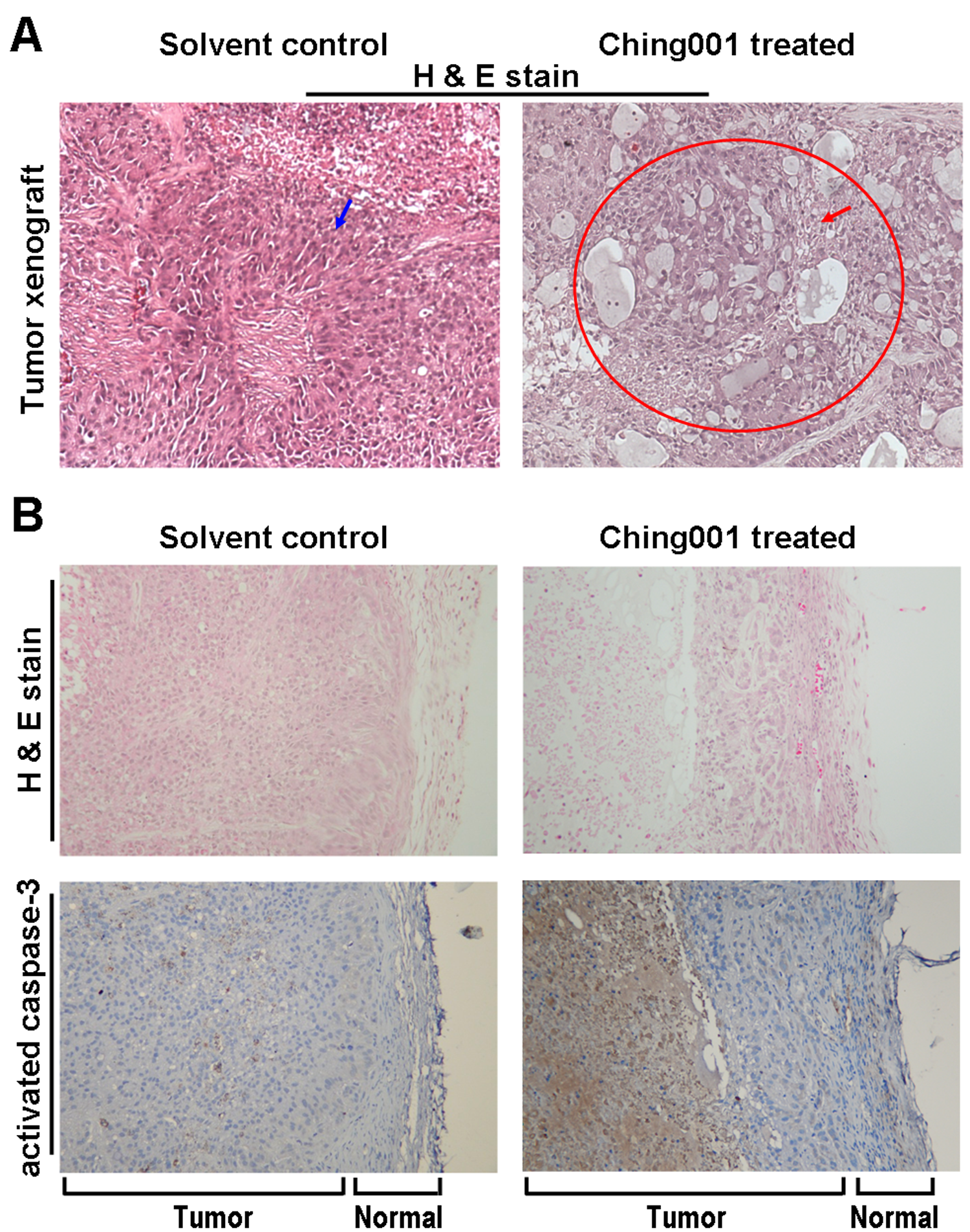

Supplement: Figure S4 — H&E-stain and activated caspase-3 IHC-stain of tumor xenograft tissues and surrounding healthy tissues. (A) Hematoxylin and eosin (H&E)-staining of solvent control treated xenograft tissue showed that the tumor cells grew well as a nodule (blue arrow). However, Ching001 treated xenograft tissue showed that the tumor cells in nodule had shrank in the nucleus (red circle) and bubbled in appearance between apoptotic tumor cells (red arrow). (B) H&E-staining (upper) and activated caspase-3 IHC-staining (lower) of surrounding healthy tissue (Normal) of solvent control treated xenograft and Ching001 treated xenograft showed that neither histological or apoptotic phenotype was observed. (TIF) [file pone.0062082.s004.tif]

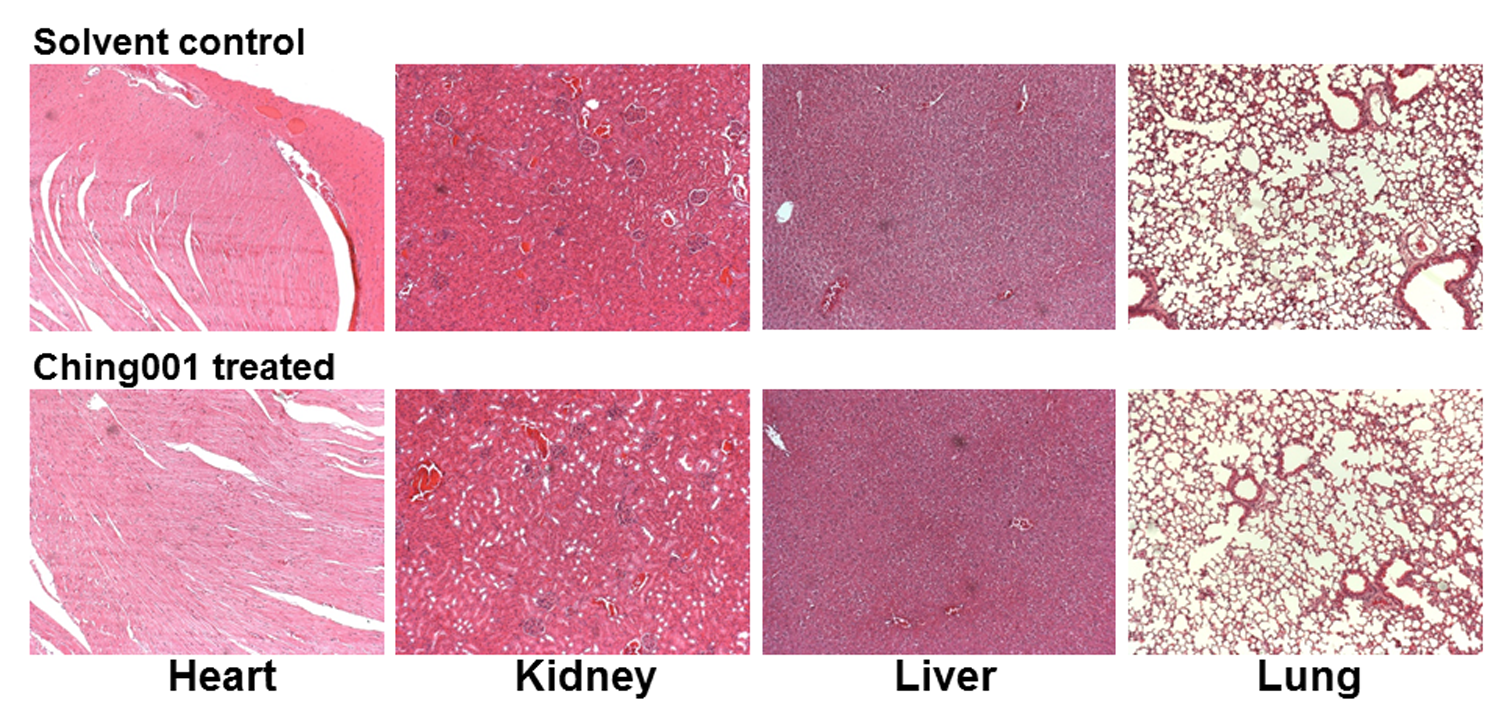

Supplement: Figure S5 — H&E-stain of major organ tissues after Ching001 treatment. H&E staining of the major organ tissues from tested ICR-nude mice with solvent control group and Ching001 treatment group. The tissues examined included heart, kidney, liver, and lung. (TIF) [file pone.0062082.s005.tif]

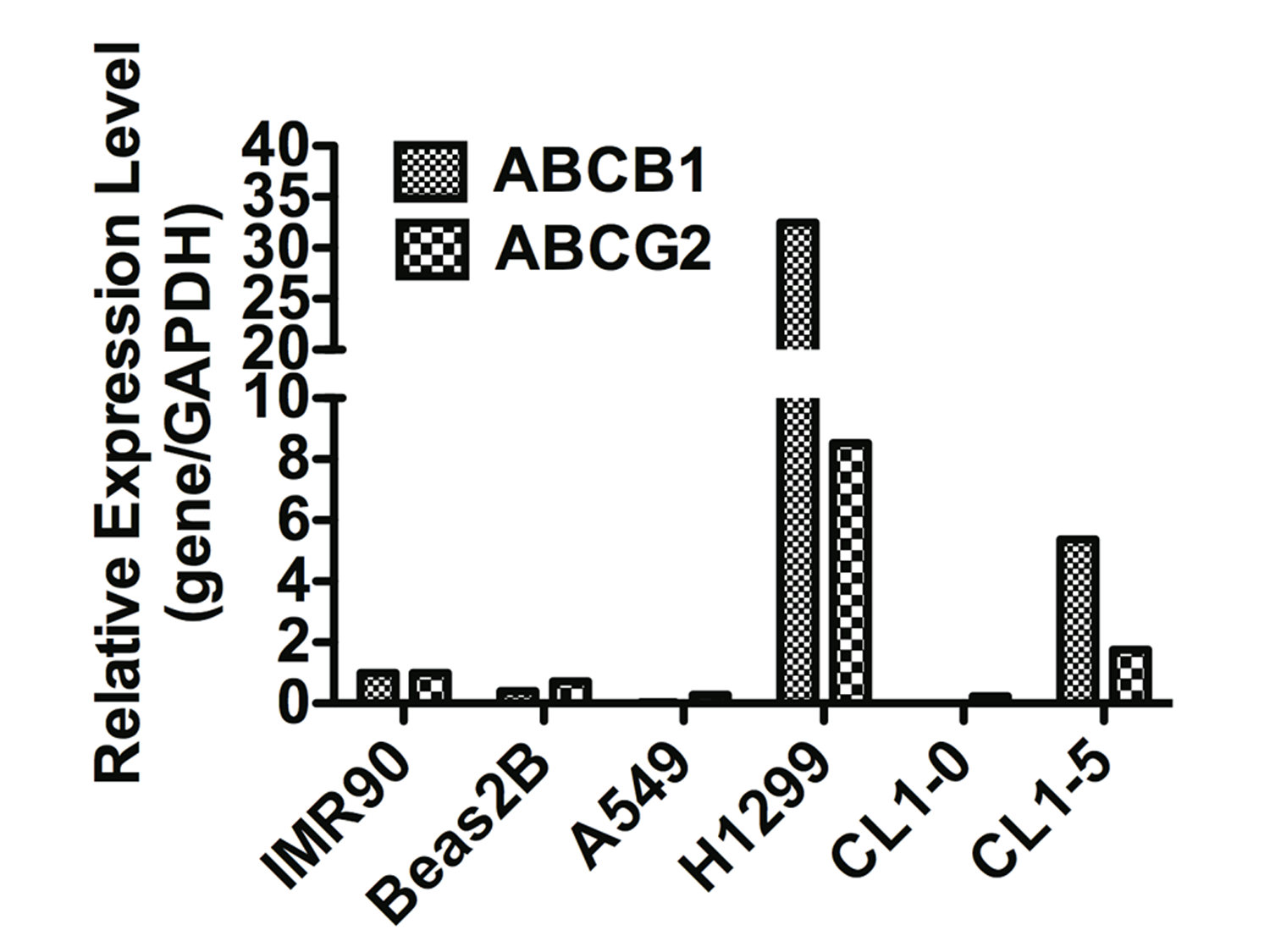

Supplement: Figure S6 — Quantitative RT-PCR analysis for relative expression of ABCB1 and ABCG2 genes encoding p-glycoproteins in various normal and cancer lung cell lines. Quantitative RT-PCR showed that the mRNA expression of ABCB1 and ABCG2 genes encoding p-glycoproteins in H1299 and CL1-5 lung cancer cells were higher than in normal lung cells IMR90 and Beas2B. (TIF) [file pone.0062082.s006.tif]

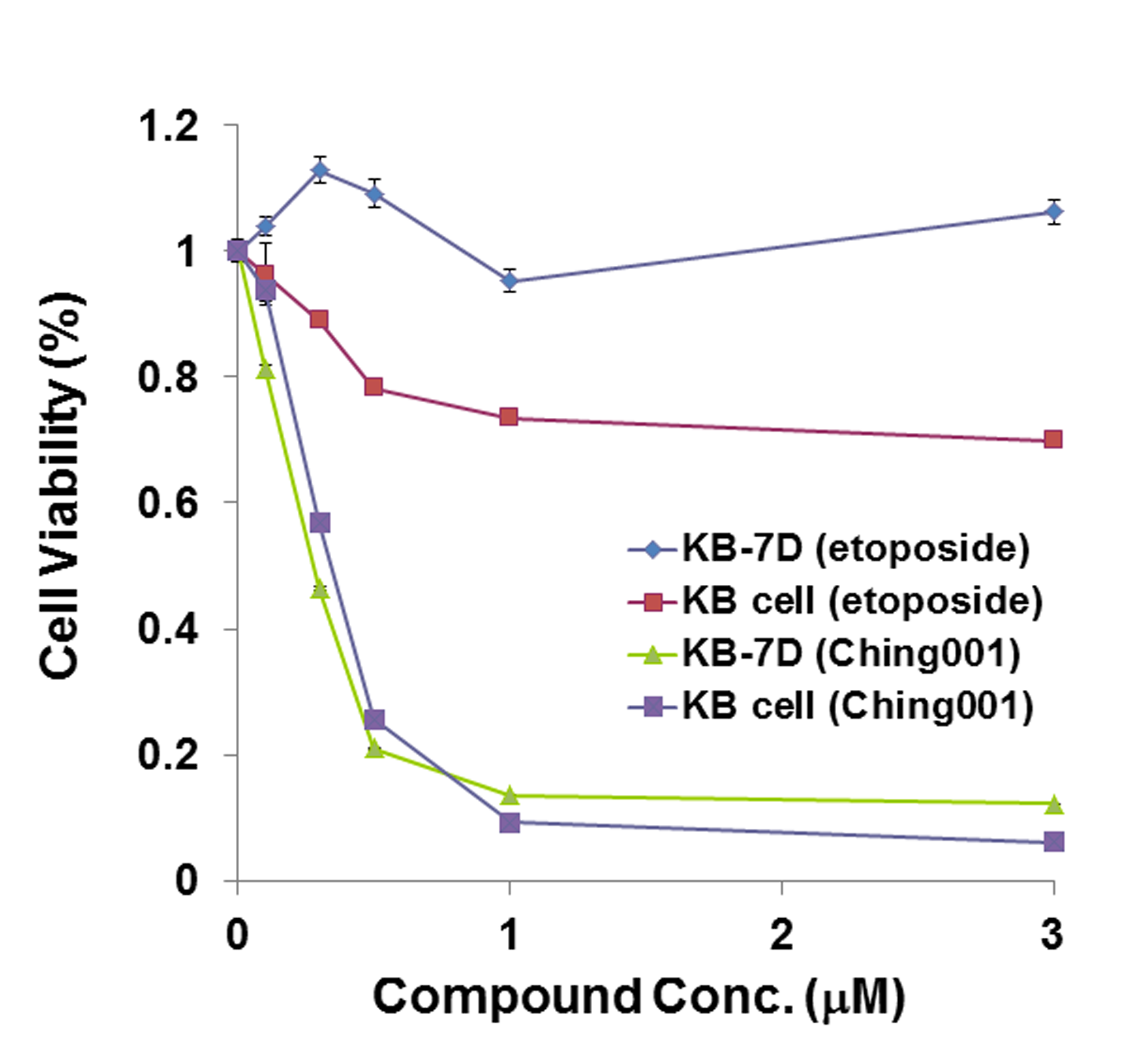

Supplement: Figure S7 — Cytotoxicity assay of Ching001 on etoposide-resistant human epidermal carcinoma cell lines. The cytotoxicity of parental human epidermal carcinoma cell line KB and etoposide-resistant KB-7D cell line was evaluated with etoposide or Ching001 treatment for 48 h. KB-7D etoposide-resistant cells did not show cytotoxicity to etoposide at all doses tested. However, Ching001 exhibited strong cytotoxicity to both KB and KB-7D cells with IC50 of 0.63 µM for KB and 0.76 µM for KB-7D. (TIF) [file pone.0062082.s007.tif]
