# Supplementary material for: A Synthetic Podophyllotoxin Derivative Exerts Anti-Cancer Effects by Inducing Mitotic Arrest and Pro-Apoptotic ER Stress in Lung Cancer Preclinical Models
Source: PLoS One. 2013 Apr 30;8(4):e62082. doi: 10.1371/journal.pone.0062082 (PMC3639983; doi:10.1371/journal.pone.0062082)
Supplement: Table S1 — Antibodies and their reaction conditions used in the present study. (DOC) [file pone.0062082.s008.doc]

**Supplementary Information**

**A synthetic podophyllotoxin derivative exerts anti-cancer effects by inducing mitotic arrest and apoptotic ER stress in lung cancer preclinical models**

**Chen et al.**

**Supplementary Table S1.** Antibodies and their reaction conditions used in the present study.

| **Target** | **K.D.** | **Raised In** | **Application** | **Dilution** | **Source** | **Catalog No.** |
| --- | --- | --- | --- | --- | --- | --- |
| α-tubulin | 55 | Mouse | WB, ICC | 1:1000; 1:200 | Millipore | 05-829 |
| β-actin | 42 | Mouse | WB | 1:5000 | Abcam | ab6276 |
| γ-H2AX | 15 | Mouse | WB, ICC | 1:1000; 1:200 | Upstate | 16-193 |
| Aurora B | 39 | Rabbit | WB, ICC | 1:1000 | Abcam | ab2254 |
| Bak | 30 | Rabbit | WB | 1:500 | Santa Cruz | sc-7873 |
| Bax | 20 | Rabbit | WB | 1:500 | Cell Signaling | cs#2774 |
| Bcl-2 | 25 | Mouse | WB | 1:1000 | Santa Cruz | sc-7382 |
| Bcl-xl | 30 | Mouse | WB | 1:1000 | Santa Cruz | sc-8392 |
| Caspase-3 | 17, 19 | Rabbit | WB, IHC | 1:1000; 1:200 | Cell Signaling | cs#9661 |
| Caspase-4 | 43, 50, 20 | Mouse | WB | 1:3000 | Sigma Aldrich | c3392 |
| DAPI | --***** | --† | ICC | 1:5000 | Sigma Aldrich | D8417 |
| eIF2α | 38 | Rabbit | WB | 1:1000 | Cell Signaling | cs#9722 |
| GADD153 | 30 | Rabbit | WB | 1:1000 | Santa Cruz | sc-575 |
| JNK | 45, 54 | Rabbit | WB | 1:1000 | Upstate | 06-748 |
| p-eIF2α | 38 | Rabbit | WB | 1:1000 | Cell Signaling | cs#9721 |
| PERK | 30 | Rabbit | WB | 1:1000 | Santa Cruz | sc-13073 |
| PI | --***** | --† | FLOW | 0.2 mg/ml | Sigma Aldrich | P4170 |
| p-JNK | 46, 54 | Mouse | WB | 1:1000 | Cell Signaling | cs#9255 |
| p-MPM2 | 97 | Mouse | WB | 1:1000 | Millipore | 05-368 |
| p-PERK | 125 | Rabbit | WB | 1:1000 | Santa Cruz | sc-32577 |
| PS | --***** | Mouse | ICC | 1:200 | Upstate | 05-719 |
| Survivin | 16.5 | Mouse | WB, ICC | 1:500; 1:100 | Santa Cruz | sc-17779 |

***** -- Molecular weight is variable.

† -- It is used for nuclear staining.
